# Heart-Healthy and Stroke-Free, 2008

**Published:** 2008-03-15

**Authors:** Darwin R Labarthe

**Affiliations:** Division for Heart Disease and Stroke Prevention, National Center for Chronic Disease Prevention and Health Promotion, Centers for Disease Control and Prevention

To celebrate the first decade of Centers for Disease Control and Prevention (CDC) support to states for heart disease and stroke prevention, it is fitting to begin with the U.S. congressional action that launched the program in 1998. The immediate antecedents of that action, its major consequences, and certain parallel developments help to put this event in perspective and make clear the pressing tasks from 2008 forward.

## 1998: Landmark Congressional Action

Legislation passed by the U.S. Congress in fiscal year 1998 was a landmark in public health efforts to improve cardiovascular health in America. This legislation is discussed in the conference report, House Report 105-390, and reads, in part, as follows:

The conferees concur with the House report language regarding the need for a comprehensive cardiovascular program, with particular emphasis on risk factors and the promotion of healthy behaviors (1).

For this purpose, Congress appropriated $8.1 million (1).

House Report 105-205 addressed the need for such a program in greater detail:

The Committee is concerned that cardiovascular disease, principally heart disease and stroke, accounts for more than 40 percent of all deaths in the United States, killing over 950,000 Americans each year. The major risk factors for cardiovascular disease are modifiable and often preventable. States receive no targeted Federal resources and many have limited resources to devote to the prevention of cardiovascular disease. An integrated, comprehensive, and nationwide program which would effectively target cardiovascular disease and its risk factors is needed. The Committee encourages CDC to begin to establish a national cardiovascular disease program. This program will provide assistance to States, support research, surveillance and laboratory capacity, and reduce risk factors for cardiovascular disease by promoting healthy behaviors. The Committee recommends that priority be given to those States with the highest age-adjusted death rates due to cardiovascular disease (2).

With the initial appropriation and the expressed will of Congress, CDC launched the National Heart Disease and Stroke Prevention Program in 1998 by providing funds to eight states to set up programs to prevent these conditions. Six states (Alabama, Georgia, Kentucky, Mississippi, Missouri, and South Carolina) were funded at the capacity-building (i.e., planning) level; two states (North Carolina and New York) were funded at the implementation (i.e., intervention) level.

## 1994: The Purple Book

Before the 1998 legislation, CDC was already engaged in some cardiovascular-related activities, such as the Lipid Standardization Program and the Behavioral Risk Factor Surveillance System. In 1988, mounting public health concern about chronic diseases — cardiovascular diseases (CVDs) being prominent among them — led CDC to establish the National Center for Chronic Disease Prevention and Health Promotion (NCCDPHP). In 1989, the Cardiovascular Health Studies Branch was established within NCCDPHP. The branch soon collaborated with the Indian Health Service in the Inter-Tribal Heart Project, which surveyed Chippewa and Menominee communities in 1991. Still, these cardiovascular activities were few in number and modest in scale.

At the time, some states were undertaking innovative activities for CVD prevention, but their capacities and resources were limited. CDC and the National Heart, Lung, and Blood Institute (NHLBI) together supported the CVD Plan Steering Committee, which guided development of the 1994 report *Preventing Death and Disability From Cardiovascular Diseases: A State-Based Plan for Action* (usually referred to as "the purple book" because of its cover art) (3). In addition to the two funding agencies, major partners in developing the plan were the Association of State and Territorial Chronic Disease Program Directors, the Association of State and Territorial Directors of Health Promotion and Public Health Education, and the Association of State and Territorial Public Health Nutrition Directors.

The common vision the partners brought to this task was described in these terms:

To improve the cardiovascular health of all Americans, every state health department will have the commitment, capacity, and resources to implement comprehensive cardiovascular disease prevention and control programs (3).

The report outlined the burden and costs of CVD, reviewed current knowledge regarding risk factors, noted state perspectives on the need for CVD prevention programs, and presented a vision of future programs and strategies to implement them.

Core functions were to be cardiovascular health-related data collection, surveillance, and outcome monitoring; public education and information dissemination; targeted outreach and linkage to services; leadership, policy development, and environmental support; and accountability and training. Strategies addressed were comprehensive; they set a broad agenda for the states regarding their organization, needed collaborations, and action areas.

Notable was a reference to the need for federal support:

. . . to provide assistance in the areas of national data assessment, coordinated technical assistance and financial support, national health promotion and disease prevention campaigns, materials and methods development and training, state-based surveillance of risk factors and policy changes, and assessment of overall program direction and impact (3).

The call for increased capacity and resources for the states and the accompanying plan to achieve improvements in cardiovascular health can reasonably be inferred to have been important contributors to Congress's decision to fund a CVD prevention program in 1998.

## Major Parallel Developments

Since 1998, significant developments paralleled implementation and expansion of the State Heart Disease and Stroke Prevention Program. Four of these developments are especially noteworthy.

- *Healthy People 2010* (4) consolidated 16 objectives for heart disease and stroke prevention into a single section (Focus Area 12) and for the first time designated CDC as co-lead agency with the National Institutes of Health. Thus CDC shares accountability for progress toward achievement of these objectives. A significant feature was the uniquely insightful statement of the goal for heart disease and stroke prevention, which made apparent the fundamental distinctions — and interrelationships — among prevention of risk factors, detection and treatment of risk factors, early identification and treatment of heart attacks and strokes, and prevention of recurrent cardiovascular events. 
- Strengthened and expanded partnerships were in large part stimulated by *Healthy People 2010* (4). The American Heart Association/American Stroke Association (AHA/ASA) adopted the *Healthy People 2010* calendar, setting their long-term strategic goals to coincide with this decennial plan. In March 2000, leaders of the AHA/ASA and NCCDPHP met in Atlanta to exchange information about strategic plans. Shortly thereafter AHA/ASA proposed to Dr David Satcher, then Assistant Secretary for Health and Surgeon General of the United States, the establishment of a formal partnership, the Healthy People 2010 Partnership for Heart Disease and Stroke Prevention. Accordingly a memorandum of understanding was signed on February 1, 2001, by representatives of AHA/ASA, CDC, NHLBI, the National Institute of Neurological Disorders and Stroke, and the Office of Disease Prevention and Health Promotion in the Office of the Secretary, Department of Health and Human Services. Later, the Centers for Medicare and Medicaid Services and the Indian Health Service became partners as well. These agencies have since collaborated on several unique activities in support of *Healthy People 2010* goals for heart disease and stroke prevention. 
- Given CDC's new responsibilities in the area of CVD, the need for a long-range strategic plan for public health approaches became apparent. Such a plan was needed to guide not only CDC but also all agencies and institutions throughout the nation involved in efforts to prevent CVD. Under the leadership of CDC, joined by AHA/ASA and the Association of State and Territorial Health Officials (ASTHO), a planning process was undertaken beginning in December 2001. That process culminated in the release by then Secretary of Health and Human Services Tommy Thompson of *A Public Health Action Plan to Prevent Heart Disease and Stroke* *(Action Plan)* in April 2003 (5).
  The *Action Plan* was the product of more than 120 volunteer members of a guiding working group, 5 expert panels, and participants in the ad hoc National Forum for Heart Disease and Stroke Prevention convened to review and comment on the draft recommendations of the plan. Especially prominent in this working partnership was the Cardiovascular Health Council of the Association of State and Territorial Chronic Disease Directors, now the National Association of Chronic Disease Directors, whose members represented state health departments. Local health departments, academic centers, the Healthy People 2010 Partnership, international organizations and agencies, and several divisions within CDC were also represented.
  This collaboration led to the establishment of the National Forum for Heart Disease and Stroke Prevention (National Forum) as the principal vehicle for long-term implementation of the *Action Plan* (5). Action priorities were set, seven implementation groups were set up as the foundation of the National Forum, and an organizational structure was adopted under the leadership of a coordinating board to represent the implementation groups, the lead partners (CDC, AHA/ASA, and ASTHO), and delegates from member organizations. The work of the National Forum is continuous, and the membership of some 90 organizations and agencies meets annually around the anniversary of the release of the *Action Plan*. 
- With growth of the National Heart Disease and Stroke Prevention Program, completion of the *Action Plan*, and establishment of the National Forum, calls intensified for creating a new division within NCCDPHP with responsibility for CDC's CVD prevention activities. The Division for Heart Disease and Stroke Prevention became official in January 2006. It was formed by combining three entities: the Cardiovascular Health Branch, Division of Adult and Community Health (DACH); the Office of the Associate Director for Cardiovascular Health Policy and Research, DACH; and the WISEWOMAN Program, Division of Nutrition and Physical Activity.

## A Decade On

Today, with the funds appropriated for fiscal year 2007, the National Heart Disease and Stroke Prevention Program supports 33 states and the District of Columbia in their activities related to heart disease and stroke prevention. Capacity has increased substantially during the first decade of the program, and experience in implementing interventions is accumulating. The contributors to this special issue of *Preventing Chronic Disease* provide numerous illustrations of the program's accomplishments to date.

Although we have had many successes, 17 states with an aggregate population of more than 50 million Americans still remain without the targeted support that this program is intended to offer. We do reach out to all state health departments and support their participation in training and related activities. But even the states that do receive program funds have insufficient capacity to meet fully the functions and responsibilities expressed in 1994 in *Preventing Death and Disability From Cardiovascular Diseases: A State-Based Plan for Action* — the purple book (3).

## Pressing Tasks

Further progress is needed to fulfill the congressional vision of a "national cardiovascular disease program" expressed in 1998. The yet-to-be-funded states are a high priority for further program development, as is enabling every state to progress toward the *Healthy People 2010* goals of preventing risk factors, detecting and treating risk factors, identifying heart attacks and strokes early, and preventing recurrent cardiovascular events. U.S. territories and tribal organizations are also in need of such support.

Fundamental to program development, implementation, and evaluation is the continuous assessment of the health of communities, states, and the nation with respect to indicators of heart disease and stroke: prevailing relevant policies; underlying social and environmental conditions; populationwide patterns of health behaviors; risk factor incidence, detection, treatment, and control; rates of major cardiovascular-related events such as heart attacks, strokes, and hospitalizations for heart failure; incidence and case fatality; and disability, dependency, quality of life, and recurrence of events for people who survive the initial episode. Monitoring these indicators among all major groups within the population is necessary to assess the burden; to recognize disparities; to develop and implement relevant policy, environmental, and systems change; and to evaluate the effectiveness of interventions in eliminating preventable cardiovascular risks and events.

For this purpose both strengthened surveillance, as recommended under the aegis of the National Forum for Heart Disease and Stroke Prevention, and thoughtful formulation of goals and objectives for *Healthy People 2020* are important (6).

Full implementation of the *Action Plan* continues to be central to our efforts, and strengthening support for the National Forum and its implementation groups is critical to the success of this effort.

We are greatly encouraged by the progress of the past decade. It strongly reflects an ever-widening recognition of the need to increase our investment in prevention, to transform public health agencies into instruments for change, and to prevent the causes of cardiovascular and other chronic diseases. At the same time, we are endeavoring to address the full spectrum of available interventions to help victims of these diseases and to protect healthy people from getting these diseases. We have much work to do. But the opportunity is immense, and we have many reasons for optimism that progress during the coming decade will surpass that of the last.
